# Survival Rate and Transcriptional Response upon Infection with the Generalist Parasite *Beauveria bassiana* in a World-Wide Sample of *Drosophila melanogaster*

**DOI:** 10.1371/journal.pone.0132129

**Published:** 2015-07-08

**Authors:** Francesco Paparazzo, Aurélien Tellier, Wolfgang Stephan, Stephan Hutter

**Affiliations:** 1 Department of Biology II, Ludwig-Maximilians-Universität München, 82152 Planegg-Martinsried, Germany; 2 Section of Population Genetics, Technische Universität München, 85354 Freising, Germany; CINVESTAV-IPN, MEXICO

## Abstract

The ability to cope with infection by a parasite is one of the major challenges for any host species and is a major driver of evolution. Parasite pressure differs between habitats. It is thought to be higher in tropical regions compared to temporal ones. We infected *Drosophila melanogaster* from two tropical (Malaysia and Zimbabwe) and two temperate populations (the Netherlands and North Carolina) with the generalist entomopathogenic fungus *Beauveria bassiana* to examine if adaptation to local parasite pressures led to differences in resistance. Contrary to previous findings we observed increased survival in temperate populations. This, however, is not due to increased resistance to infection *per se*, but rather the consequence of a higher general vigor of the temperate populations. We also assessed transcriptional response to infection within these flies eight and 24 hours after infection. Only few genes were induced at the earlier time point, most of which are involved in detoxification. In contrast, we identified more than 4,000 genes that changed their expression state after 24 hours. This response was generally conserved over all populations with only few genes being uniquely regulated in the temperate populations. We furthermore found that the American population was transcriptionally highly diverged from all other populations concerning basal levels of gene expression. This was particularly true for stress and immune response genes, which might be the genetic basis for their elevated vigor.

## Introduction

Heritable variability within a species provides the basis for natural selection to occur. This fundamental principle of theoretical evolutionary biology has prompted many studies focusing on the surveillance of variability in natural populations. A key expectation of natural selection acting on a species, which is distributed over a wide range of heterogeneous habitats, is that of local adaptation. That is, populations from different environments would exhibit different phenotypic optima and/or fix genetic variants that provide increased fitness in the context of their local habitat. This will ultimately lead to an observable differentiation between populations on the genotypic and phenotypic level, as long as gene flow is limited [1]. A phenotype of major importance in this respect is resistance to infection by pathogens. Pathogens, or parasites, depend on their host for growth and reproduction and are at the same time a source of selection on their host for reduced damage and/or clearance. The ability to resist a parasite increases host fitness, because it lowers the probability of infection, and in case of successful infection reduces damages. In the presence of pathogens it is thus expected that there is selection for increased resistance in hosts [2]. However, in the absence of pathogen pressure, other traits, such as survival, fecundity, competitiveness are more important. As each of these traits as well as defense response bears different metabolic costs, the level of resistance to pathogen is the result of a trade-off between conflicting needs [3].

The strength of the defense is usually modulated by a complex multi-gene response in a quantitative manner [4]. Understanding the evolutionary forces and genetic underpinnings of local adaptation in host and parasite populations are key questions in evolutionary genetics, namely to uncover trade-offs between specificity, quantitative defense pathways, and other host life-history traits. An additional complexity lies in the spatial structuring of host and parasite with spatially heterogeneous strength of parasite pressure (for example, parasite prevalence). Variable dynamics of reciprocal adaptation at the phenotypic and genotypic levels, and different trade-offs among populations are expected and observed as described in the so-called “geographic mosaic of coevolution” [5].

The fruit fly *Drosophila melanogaster* is an excellent model organism for studying both species-wide genetic and phenotypic variability. Although it originated in sub-Saharan Africa it can now be found world-wide, inhabiting diverse habitats [6]. Populations have been found to differ significantly at the phenotypic [7,8], genetic [9–11] and gene expression levels [12,13]. The distribution in tropical as well as temperate climatic regions makes this host species particularly interesting for studying the evolution of resistance to parasites. A general effect of latitude has been claimed to influence host-parasite interactions. Due to the higher species richness at low latitudes, parasite prevalence and therefore host investment in immune defense should be higher in tropical compared to temperate populations. Evidence for this hypothesis exists in few species, although some exceptions have also been reported [14].

In order to test this case of heterogeneous coevolution in space, a previous study [15] found variation in survival of *D*. *melanogaster* to infection with the entomopathogenic fungus *Beauveria bassiana* both within and between populations. A higher survival in Afro-tropical host populations compared to temperate ones (originating from Europe and North America) was reported. This last finding supports the theory of higher immune investment in the tropics, and thus local adaptation for the evolution of defense response. Given that *B*. *bassiana* has a broad host range it seems, however, unlikely that it is engaging in a strict co-evolutionary arms-race with a particular host species or population [16]. Therefore the *D*. *melanogaster*-*B*. *bassiana* system presents the possibility to study how the host responds to a generalist fungal pathogen and to assess if variability among host populations is present for specificity and resistance, possibly due to different life history strategies. It is unclear, moreover, if the pattern found by Tinsley *et al*. [15] holds on a global scale or if it is simply a feature of the populations tested. Previous studies in birds have found conflicting patterns, where for some species immune response was more pronounced in the tropics [17], while for others this was more the case in temperate populations [18]. In our study we therefore assessed resistance to infection with *B*. *bassiana* in four additional *D*. *melanogaster* populations, including a tropical one from Asia, a region that has not been investigated up to now.

In addition to mortality levels we also wanted to gain insight into the actual immune investment of the populations and uncover potential differences between them. We therefore quantified transcriptional response to infection in our world-wide sample using RNA-seq. Given that the expression of resistance genes forms the basis of the immune response in a host species, differences in expression profiles between hosts from different localities might represent the outcome of local selection. Two previous studies have already described gene expression changes in *D*. *melanogaster* in response to infection with *B*. *bassiana* using microarray approaches. De Gregorio *et al*. [19] surveyed the transcriptional response at four time points, from twelve to 96 hours after infection. Among the genes that displayed a significant change in gene expression 32 were already known as immune genes, while 368 had previously not been associated with immunity. This exemplifies that transcriptome analysis can also help us to identify new candidate immune genes and get a better understanding of how immune response unfolds. Roxström-Lindquist and collaborators [20] assessed gene expression 24 hours after infection by *B*. *bassiana*, the protozoan parasite *Octosporea muscae domesticae*, the Gram-negative bacterium *Serratia marcensens* and *Drosophila* C virus. They found a high degree of microbe specificity, with the fungal infection generating the strongest response, with 298 genes induced.

A common characteristic of the studies cited above is that only a single inbred lab strain of *D*. *melanogaster* was used (*Oregon-R* or *Canton-S*), both of which are derived from North America. This provides us only with a limited view of the outcome of host-parasite interaction and host transcriptional response in terms of potential population differences. Nevertheless, different genes were found to be induced after *B*. *bassiana* infection in De Gregorio *et al*. [19] and Röxstrom-Lindquist *et al*. [20]. This hints toward genetic variability affecting host immune response, although discrepancies in the infection procedure and in data analysis could also play a role. A potential caveat of the aforementioned studies is the use of inbred lines. It has been shown that inbreeding reduces fitness and resistance to parasites in *D*. *melanogaster* [21]. The observed immune responses might therefore not be indicative of the situation in natural populations.

In our study we therefore created out-bred populations of flies from two temperate and two tropical localities. These were then used for infection experiments with *B*. *bassiana* and survival rates and transcriptional response in the hosts was measured at two time points. Early response to infection has been identified as an important predictor of survival ability in *D*. *melanogaster* [22]. There is evidence that *D*. *melanogaster* changes in gene expression occur already few hours after *B*. *bassiana* infection [4,19]. On this basis we choose two time points for our analysis: eight and 24 hours post infection.

## Materials and Methods

### Biological material

*D*. *melanogaster* samples were derived from established lab stocks and were provided by the labs that performed the collections. No animal collections were carried out by the authors. Infection experiments were performed on *D*. *melanogaster* lines derived from four localities: Lake Kariba, Zimbabwe [23], Leiden, the Netherlands [24], Kuala Lumpur, Malaysia [25] and Raleigh, North Carolina, USA [26]. These populations were chosen to represent major geographic regions in the world-wide distribution of *D*. *melanogaster* [27], from temperate (Leiden and Raleigh) and tropical climates (Lake Kariba and Kuala Lumpur). Flies were reared at 23°C on standard fly media with a 14 hours light and 10 hours dark cycle.

In order to avoid the effects of inbreeding on fitness and resistance to parasites [21], out-crossed populations for each of the four localities were created. Twelve inbred lines from each population (ten in the case of the African population) were reciprocally crossed in six (or five) pairs to obtain a set of twelve (or ten) heterozygote F1 flies. 50 males and 50 females of each F1 line were then pooled in population cages. These populations were then left to mate randomly. In order to maintain these out-crossed populations at a constant size of around 1,000 flies, the appropriate number of eggs was collected every two weeks and a new generation was started following Clancy and Kennington [28]. A second replicate population cage for each of the four localities was set up after several generations of inbreeding by splitting up the original population. This second cage is used as a replicate to control for the effect of drift inherent to fixed population sizes in laboratory conditions.

The *B*. *bassiana* strain labeled 1630 (collected in France in 1984) was used for infection experiments. It stems from the Collection of Entomopathogenic Fungi of the United States Department of Agriculture [29]. To overcome any attenuation of virulence that may have occurred during maintenance in the lab, the strain was passed through a *Drosophila yakuba* lab strain following Tinsley *et al*. [15]. This allowed us to increase the virulence of the strain, while avoiding fungal adaptation to a specific *D*. *melanogaster* line. Flies were infected by spraying with a fungal spore/oil suspension and sporulating cadavers were collected during a ten-day period. Cadavers were subsequently homogenized in Shellsol T oil and plated onto potato dextrose agar containing chloramphenicol (5 x 10−^5^ g/ml) to grow the fungus and confirm the death by infection. Plates were incubated for two weeks at 25°C in complete darkness and then dried at room temperature for one week. Sporulating material was collected from each plate, dried in silica gel and stored in the fridge suspended in oil (87.5% Shellsol T, 12.5% Ondina El).

### Infection procedure

*B*. *bassiana* spore concentration was adjusted to 10^8^ spores/ml by adding the appropriate volume of oil. The suspension was agitated using a probe sonicator to avoid spore clustering and concentrations were confirmed using a haemocytometer. The suspension was then applied to transparency film using an airbrush and dried for one to two weeks in a dark closed cabinet to avoid spore decay due to UV light. The homogeneity of the spore spray on the film was controlled by visual inspection. Stripes of 5 cm width were then cut and inserted into standard *Drosophila* vials to cover the walls. For control vials this process was repeated with oil not containing spores (so-called mock control). Before each round of infection, spores were set in potato dextrose agar medium for 24 hours to test their ability to germinate. Observed rates of germination were over 90%. For infection, three to five days old male flies were collected and placed in vials with standard food. After an additional one to three days 15 flies were transferred to either vials containing transparency film with the spore suspension or control vials containing only oil. Flies were exposed for three days to the infection/control vials before being transferred to standard vials without transparency film.

Every three days flies were transferred into fresh standard vials and the number of dead flies was recorded. Other possible causes of loss of flies independent of infection or natural mortality, such as escaped flies, were reported. During the course of the infection experiment only fly food devoid of any anti-fungal/anti-bacterial preservatives such as Nipagin or propionic acid was used. Vials were inspected daily for contamination with unknown bacteria or molds and flies were immediately transferred into fresh vials in such cases. Vials that contained severe contamination were completely discarded. The experiment was carried out at 25°C and a 14h/10h light/dark cycle. Fly carcasses were monitored for the outgrowth of *B*. *bassiana* during the experiment in order to confirm a successful infection and such outgrowth was consistently observed in infected flies. Infection experiments for all four populations were carried out three times: Twice to assess mortality over a three-week period and once for profiling gene expression in the early stages of infection.

### Mortality analysis

Fly mortality was analyzed using generalized linear mixed models (GLMM) [30]. Experimental variables such as treatment (infection or control), time after infection, sex, population, and replicate cage were used as covariates, as well as the interaction between treatment and population. The individual vial was used as a random factor to account for the repeated measurements from the same vial.

When assessing mortality rates over time we specified multiple time covariates because we did not make any assumption on how time affects mortality. To determine which time covariate or combination of time and covariates best described the data, a likelihood ratio test was performed [31]. We additionally used survival analysis methods where the time to death of each single fly is used as dependent variable and the effect of covariates on survival time is assessed [32]. For this, Cox proportion hazard models [33] were employed and the data is graphically represented using the Kaplan-Meier estimator [34]. All analyses were performed in R [35] using the libraries lme4, coxme and survival.

### Gene expression profiling

In order to assess transcriptional response to infection total RNA was extracted from flies that had undergone three different treatments: Control flies that had undergone an eight-hour mock-infection (see above), flies that had been exposed to fungal spores for eight hours and flies that had been exposed to fungal spores for 24 hours. Total RNA was extracted from 30 male flies from each of the four populations using the Master Pure RNA Purification kit (Epicentre, Madison, WI). Each extraction was performed three times independently leading to a total of 36 samples.

RNA samples were sent to GATC Biotech (Konstanz, Germany) for quality control and sequencing. One American control sample showed RNA degradation and was removed from further analysis. For the remaining samples mRNA was isolated, retro-transcribed and sequenced on an Illumina HiSeq 2000 sequencing machine producing 50nt single-end reads. The reads were mapped to the *D*. *melanogaster* reference transcriptome release 5.57 [36] using NextGenMap [37]. Reads that mapped to transcripts belonging to the same gene were summed up and the resulting gene counts were used for further analysis. Raw read files and gene counts were submitted to the NCBI Gene Expression Omnibus [38] under series GSE67177.

Principal component analysis (PCA) and differential gene expression analysis were performed using R [35] and the DESeq2 package [39]. The data set was split up to contrast expression differences to the controls at the eight- and 24-hour time points separately. Differential expression was analyzed by fitting a generalized linear model with two factors (population and infection treatment). The general effect of infection was analyzed by contrasting the treatment effect of infected flies versus control flies. Genes that show expression differences with a p-value below 0.05 after adjustment for multiple testing were considered significant. Population specific responses to infection were uncovered by fitting a second model that included the interaction term of population and treatment and searching for significant interactions. Finally, we searched for genes that were generally differentially expressed between populations, irrespective of infection status. For this a model including interactions was fitted to the complete data set and each population was contrasted with all other three populations combined.

For analysis of gene function gene ontology (GO) terms for individual genes were extracted from FlyBase [36]. Systematic over-representation of functional terms for gene sets was analyzed with GOrilla [40] by contrasting the target gene list with the list of all genes in the genome. GO terms with corrected *q*-values [41] below 0.05 were considered significant.

## Results

### Geographic variation of infection resistance

We performed two independent infection experiments using the *B*. *bassiana* strain 1630 (hereafter labeled replicates 1 and 2). We assessed mortality three days after treatment and found that control flies had a significantly higher mortality rate than infected flies. This counterintuitive result was consistent in both replicates (Table 1). The higher mortality was not related to fungal infection, but a consequence of control flies getting stuck to the vial walls or soaked into the oil. This was due to an increased viscosity of the pure oil as compared to oil containing spores. After three days we also observed variability in mortality among populations in infected and control vials (Table 1). This reflects a difference in general vigor, which is a flies' capability to cope with stress, with temperate populations showing higher vigor than tropical populations. For replicate 1 this effect was statistically significant when American and European flies were compared to the African population using GLMM (*p* = 0.001 and *p* = 0.02 for America and Europe, respectively).

**Table 1:** Mean mortality rates (and SD) in percent three days after infection for male *D*. *melanogaster* from four populations.

	Replicate 1		Replicate 2	
Population	Infected	Control	Infected	Control
Europe	8.6 (10.7)	16.7 (14.4)	26.5 (23.4)	27.8 (15.1)
America	7.7 (8.6)	12.8 (12.8)	11.6 (10.4)	18.3 (14.8)
Asia	12.9 (12.7)	17.3 (11.2)	33.4 (20.9)	31.3 (25.0)
Africa	14.8 (14.1)	19.0 (14.5)	30.0 (20.7)	39.9 (16.2)

**Fig 1:**
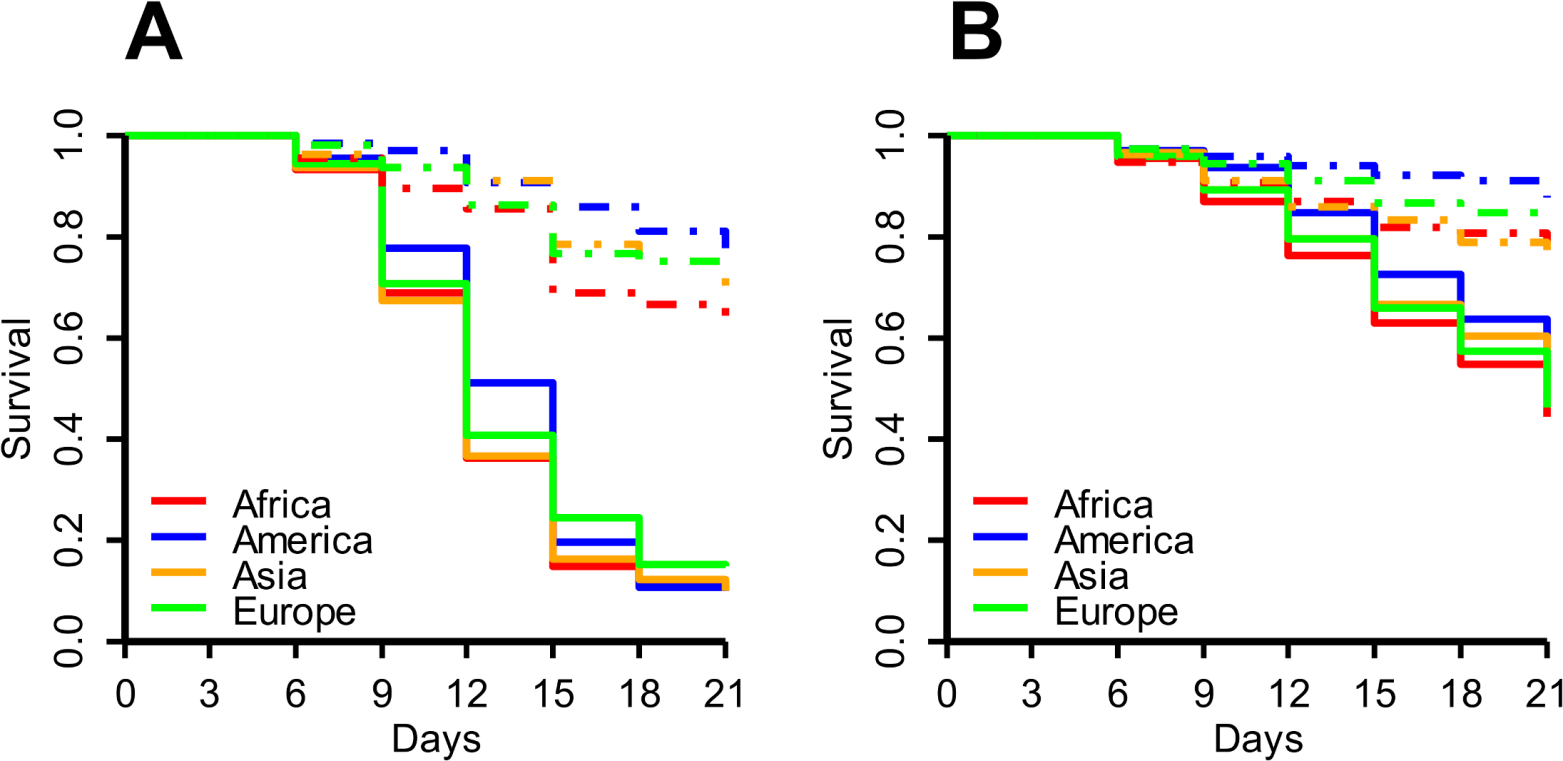
Survival after infection. Kaplan-Meier plots for the survival of flies from the four populations in replicates 1 (A) and 2 (B). Flies from uninfected control lines are represented by dashed lines; flies exposed to *B*. *bassiana* are represented by solid lines.

To account for the effect of oil, mortality was assessed starting six days after infection when flies were transferred to standard vials, using the survivors from the previous vials as the baseline (see Materials and Methods). For all populations we observed a clear effect of increased mortality upon fungal infection in both replicates (Fig 1), as would be expected. The effect was significant in our analysis using GLMM (*p* = 0.0005, *p* = 0.002 for replicates 1 and 2, respectively), as well as Cox hazard models (*P* < 10^−13^, *P* < 10^−6^ for each replicate, respectively).

Furthermore, we could confirm the trend of increased vigor in flies from temperate populations, specifically from America, which is visible as increased survival in the control vials compared to tropical flies (Fig 1). In replicate 1 mortality was significantly lower in America compared to Africa independent of infection status (Cox hazard model: *p* = 0.023). The same was found in replicate 2 (GLMM: *p* = 0.047, Cox hazard model: *p* = 0.043).

In none of the experiments did we observe a significant effect of the population on infection induced mortality rates. In other words, no significant differences exist in resistance between *Drosophila* populations. These results do not support any differences in susceptibility to fungal infection among latitudes as previously suggested [15].

### Gene expression profiling

**Fig 2:**
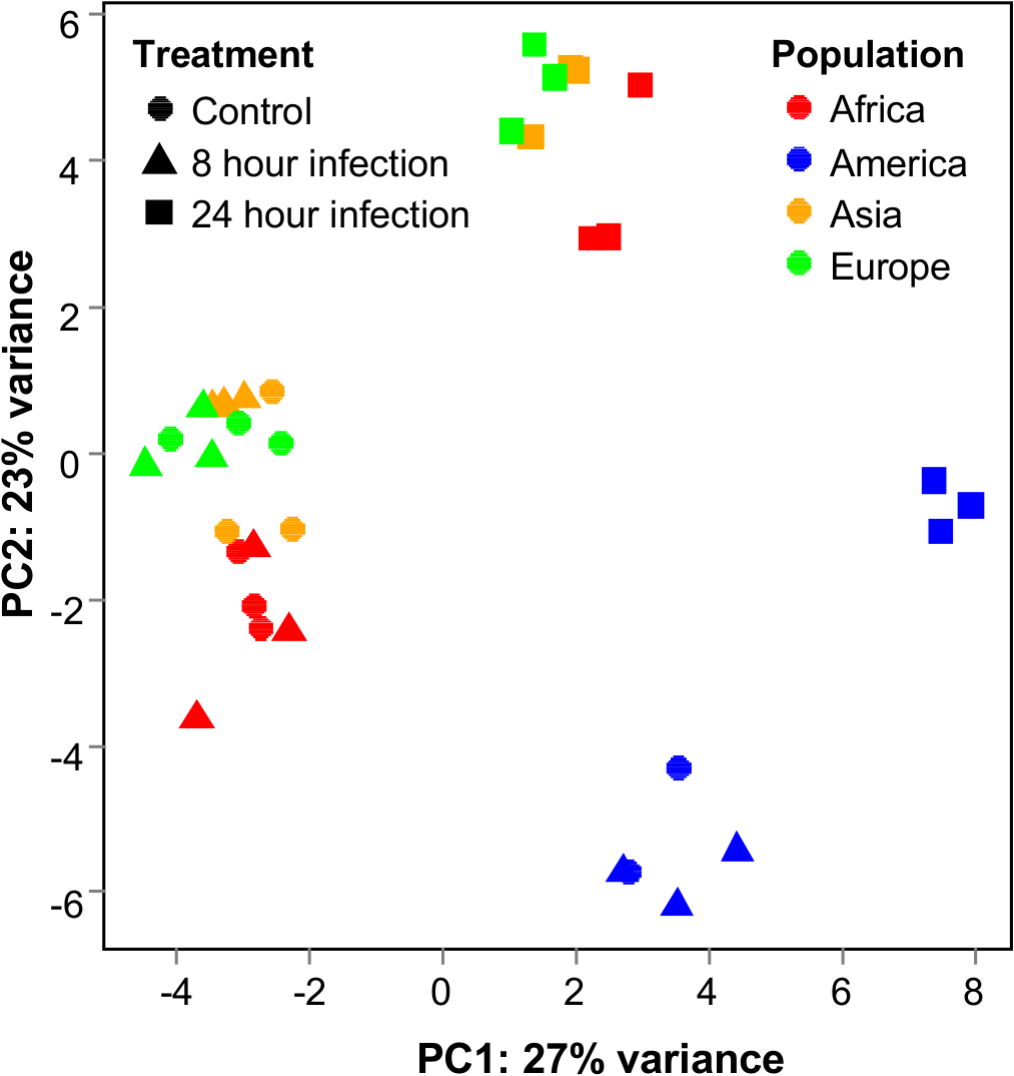
PCA plot. Principle component analysis of gene expression profiles of different treatments and populations of origin.

The 35 RNASeq libraries that passed quality control contained on average 21.9 million reads. 91.1% of these reads could be mapped to the *D*. *melanogaster* reference transcriptome, indicating that our data set is of high quality. The PCA of the transcriptional profiles shows that flies can be distinguished on the treatment and population level (Fig 2). Looking at the infection status we can see a clear separation between flies that were infected for 24 hours and all other samples. This indicates a shift in the transcriptional profile as a result of infection. Samples that were taken only eight hours after infection on the other hand cluster closely together with the controls, indicating only minor differences in expression.

Using a generalized linear model as implemented by DESeq2 [39] we could identify 20 genes that have commonly changed expression in all four populations eight hours after infection (Table 2). This rather low number suggests that the infected flies are in the very early stages of infection response where only very few genes react to the challenge of parasitic infection. However, some functional classes of genes stand out in this list. First, we find three oxidoreductases of the cytochrome P450 (Cyp450) family (*Cyp6d2*, *Cyp6g2* and *Cyp6a8*) which all show over-expression in infected flies. Furthermore, the gene *CG2064* that is also associated with oxidoreductase activity was over-expressed. Second, four genes have transferase functions (*GstD7*, *CG5999*, *Ugt36Ba*, *CG10182*). Finally, we find genes with transporter functions (*Smvt*, *List*, *CG15221*, *CG30272*). These three classes of genes have been linked to detoxification processes supporting the importance of detoxification early in infection (see Discussion).

**Table 2:** Genes with differential expression eight hours after infection over all populations.

Gene name (short name)	fold-change	Adjusted *p*-value
*Cyp6d2*	1.40	<0.0001
*CG2064*	1.25	<0.0001
*Glutathione S transferase D7 (GstD7)*	1.19	<0.0001
*CG10467*	0.88	0.0006
*CG5999*	1.17	0.0006
*Ugt36Ba*	1.18	0.0006
*Sodium-dependent multivitamin transporter (Smvt)*	1.17	0.0008
*Lithium-inducible SLC6 transporter (List)*	1.15	0.0009
*CG10182*	1.13	0.0012
*CG33012*	1.17	0.0013
*Cyp6g2*	1.14	0.0037
*Jonah 65Aii (Jon65Aii)*	1.16	0.0051
*Outer segment 4 (Oseg4)*	1.16	0.0070
*Ribonucleoside diphosphate reductase large subunit (RnrL)*	1.16	0.0089
*lysosomal enzyme receptor protein (Lerp)*	0.89	0.0089
*CG13160*	1.14	0.0095
*CG15221*	1.15	0.0137
*Cytochrome P450-6a8 (Cyp6a8)*	1.14	0.0166
*CG30272*	1.14	0.0166
*CG3635*	0.87	0.0270

In order to find genes that are regulated in a population specific manner we searched for genes that showed a significant interaction between population and infection treatment in our generalized linear model. No such genes were found for the African, American or European populations, but in the Asian population eleven genes showed significant population specific behavior eight hours after infection (Table 3). These genes have various functions, including protein binding and several metabolic activities, but no overarching common function could be found.

**Table 3:** Genes with Asian-specific effects eight hours after infection.

Gene name (short name)	Population effect (fold-change)	Adjusted *p*-value
*Allatostatin C receptor 1 (AstC-R2)*	1.48	0.0004
*CG4341*	1.27	0.0404
*Galactose-specific C-type lectin (Lectin-galC1)*	0.74	0.0442
*mushroom bodies tiny (mbt)*	1.23	0.0442
*CG18609*	0.77	0.0442
*CG8929*	1.18	0.0442
*lethal (3) 72Dr (l(3)72Dr)*	0.77	0.0442
*Fumarylacetoacetase (Faa)*	0.80	0.0463
*dalao*	1.29	0.0463
*CG8273*	1.22	0.0463
*CG42798*	0.75	0.0463

It should be noted that quantitatively the observed expression differences are generally rather small. The most extreme cases in the list of genes that were commonly induced/repressed show expression differences of around 40% (Table 2), which is similar to the population specific effect in the Asian flies (Table 3).

We repeated the analysis for transcriptional changes 24 hours after infection. Across all populations we found a total of 4,259 genes which show expression differences at the 0.05 significance level (S1 Table). This is expected, given the large shift in transcriptional profiles 24 hours after infection (Fig 2). Additionally, these expression changes are larger than those induced after eight hours. Interestingly from a functional perspective, we found that the four genes that show the largest repression (*timeless*, *vrille*, *period* and *PAR-domain protein 1*) are all involved in circadian behavior. We continued with a more systematic search for over-represented GO terms in the list of the 100 most repressed genes and found that lipase activity was enriched with seven genes being annotated for this function (*q* = 0.004). For the most strongly induced genes we did not find as clear of a pattern. However, the genes *CG17560*, *CG17562* and *CG13091* (which are the first, third and tenth most strongly induced genes) are all annotated as fatty-acyl-CoA reductases. For the list of the 100 most over-expressed genes a GO term analysis revealed that almost half of them (49 genes) have catalytic activity, which is a strong enrichment (*q* = 0.006). Among these, the glucuronosyltransferases are represented by five genes (*q* = 0.007). Of the genes that are classically thought to be immune related one group stands out in the list of top 100 over-expressed genes, which is the *immune induced molecule* gene family. It is represented with five members (*IM1*, *IM2*, *IM3*, *IM10* and *IM23*). Moreover, we find that two additional genes that are involved in circadian rhythm (*cryptochrome* and *clock*) are among the top 100 genes with increased expression.

**Fig 3:**
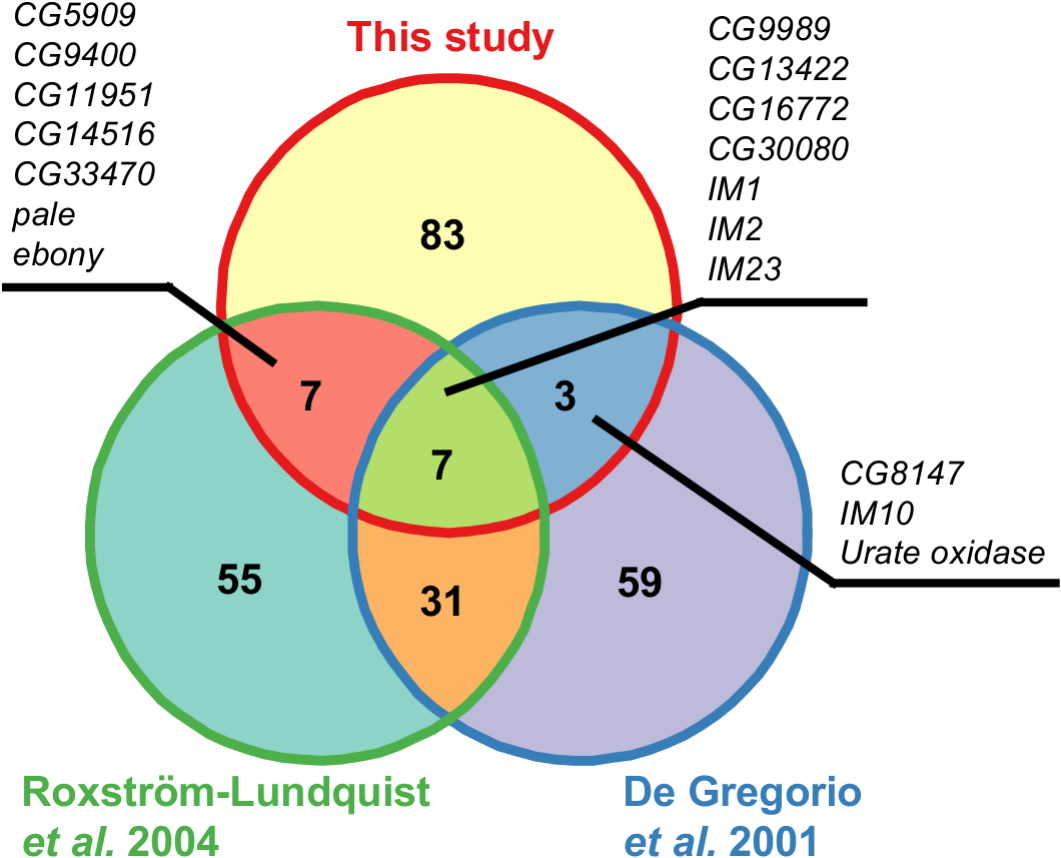
Comparison of strongly induced genes. Overlap of the 100 most strongly induced genes 24 hours after infection in this study compared to those found in De Gregorio *et al*. [19] and Roxström-Lundquist *et al*. [20]. Gene names of the genes found in the microarray studies were updated to reflect the genome annotation of Flybase release 5.57 [36].

We then compared our list of strongly induced genes with those found in previous microarray studies at the same time point (Fig 3). We find that several genes overlap between the studies, especially those belonging to the *immune induced molecule* gene family. However, overall the proportion of overlapping genes is rather small. Also, several genes that were found to be strongly induced in both microarray studies (*e*.*g*. *Drosomycin*, *Metchnikowin*) were not found to be induced in our experiments. The overlap is even smaller when looking at genes that are strongly repressed. Here we found that only a single gene, the odorant-binding protein *Obp99b*, was shared between our study and the work of Röxstrom-Lundquist *et al*. [20], while the genes *lectin-21Cb* and *CG18179* overlapped with the data set of De Gregorio *et al*. [19].

We continued to search for genes that are differentially regulated in a population-specific manner 24 hours after infection in our experiment. We found no such genes in the African or Asian populations. However, three genes in the European and 28 genes in the American population show differential expression patterns (Table 4). Two genes in the European population belong to the Cyp450 family. The gene *Cyp4e3*, which does not show expression changes over all populations, is repressed significantly in Europe. On the other hand, *Cyp4d14* is repressed in all other populations, but does not show an expression change in Europe (as a consequence of the positive population-specific effect presented in Table 4 canceling out the general negative effect over all populations). For the American population we find that the gene that is generally repressed the most (*timeless*) has a positive population effect. This means that while the gene is still repressed in American flies, the effect is less pronounced. Interestingly, we see a similar effect for *CG17560*, which is among the genes with the largest over-expression over all populations (see above). Here, while the expression change remains significant in America, it is less pronounced. We also find genes for which the general expression change is amplified within the American population (*e*.*g*. *Npc1b* and *Orct2*). And finally we find genes that change their expression specifically in the American population (*e*.*g*. *CG31233* and *CG6770*) and the reverse case where genes that are generally induced or repressed do not change their expression levels in the American population (*e*.*g*. *CG8834* and *Sirup*).

**Table 4:** Genes with European and American-specific effects 24 hours after infection.

Gene name (short name)	Population effect (fold-change)	Adjusted *p*-value	General expression change^1^	Population expression change^2^
**European population**				
*Cytochrome P450-4e3 (Cyp4e3)*	0.72	0.0112	0	-
*adenosine 3 (ade3)*	0.75	0.0122	-	-
*Cyp4d14*	1.27	0.0325	-	0
**American population**				
*CG8834*	0.67	<0.0001	+	0
*timeless (tim)*	1.47	<0.0001	-	-
*CG17560*	0.68	<0.0001	+	+
*Niemann-Pick type C-1b (Npc1b)*	0.69	<0.0001	-	-
*CG11796*	1.28	0.0001	+	+
*CG31233*	0.73	0.0003	0	-
*CG13607*	0.80	0.0014	0	0
*CG2930*	0.82	0.0025	+	0
*Organic cation transporter 2 (Orct2)*	0.76	0.0028	-	-
*Starvation-upregulated protein (Sirup)*	1.27	0.0029	-	0
*CG3106*	0.73	0.0033	-	-
*CG15263*	1.31	0.0076	0	0
*CG5724*	1.32	0.0150	+	+
*Chorion factor 2 (Cf2)*	0.78	0.0153	0	0
*CG17752*	0.77	0.0153	+	0
*CG11892*	0.77	0.0158	-	-
*goliath (gol)*	1.20	0.0173	-	-
*CG8773*	0.79	0.0293	0	-
*Hemolectin (Hml)*	0.79	0.0325	+	0
*CG6770*	1.23	0.0325	0	+
*CG10657*	0.79	0.0332	-	-
*Ecdysone-induced protein 28/29kD (Eip71CD)*	0.78	0.0334	-	-
*CG42402*	0.79	0.0395	0	0
*slimfast (slif)*	0.84	0.0480	+	0
*CG4562*	0.82	0.0480	-	-
*Phosphatidylethanolamine-binding protein 1 (Pebp1)*	0.77	0.0480	0	-
*CG30431*	0.86	0.0480	-	-
*Cyp12d1-p*	1.29	0.0480	-	0

^1^ + or–symbols indicate significant over- or under-expression (*p* < 0.05) over all populations combined, 0 indicates no expression change (see also S1 Table)

^2^ + or–symbols indicate significant over- or under-expression (*p* < 0.05) within the specific population (European or American), 0 indicates no expression change

In a final analysis we searched for genes, which show expression divergence between populations at the basal level, *i*.*e*. irrespective of infection status. We already see in the PCA (Fig 2) that the European and the Asian populations show very similar transcriptional profiles and both cluster closely with the African population. The American population, however, seems to be transcriptionally diverged form the other three populations. In order to find out which genes drive this expression divergence we searched for significant population effects in our generalized linear model. Here, we contrasted each of the four populations with all other three combined. Using this approach we find that the African population shows 1,546 genes, which are differentially expressed compared to all other populations, the Asian population shows 1,792 genes, the European population 2,089 genes and the American population 3,459 genes (S2 Table). As expected the American population shows the largest number of transcriptionally diverged genes.

To gain insight into the nature of the genes that differ in expression between populations we performed a GO analysis of the top 100 over- and under-expressed genes for each population. For the African population many genes with taste receptor activity are expressed at a lower level compared to all other populations (*q* = 0.0003). This is entirely driven by seven members of the *gustatory receptor* (*Gr*) gene family. More generally, genes with transmembrane signaling activity are under-expressed in Africa (12 genes, *q* = 0.0019). Additionally, three *larval serum protein* genes fall into this category. These genes have nutrient reservoir activity (*q* = 0.0024). For the genes which are highly over-expressed in Africa no over-represented functions could be found. However, it is interesting to note that the gene with the largest amount of over-expression is a Cyp450 gene (*Cyp313a3*), while the second and third most extreme genes (*CG33986* and *CG13675*) are involved in chitin binding. For the European population we did not find any specific functional category of genes to be systematically under-expressed. On the other side many genes with alpha-mannosidase activity are over-expressed (four genes, *q* = 0.0056). For American flies we see under-expression of serine-type endopeptidases (eleven genes, *q* = 0.0009) and structural constituents of the cuticule (eight genes, *q* = 0.0033). Many genes with transmembrane signaling activity are over-expressed in America (13 genes, *q* = 0.0018) with the *odorant receptor* (*Or*) gene family (five members) standing out and the *gustatory receptor* (*Gr*) gene family also being represented with three genes. Surprisingly, even though not captured by any specific GO term, many genes that are associated with stress or immune response were found to be generally over-expressed in American flies. This includes four Cyp450 genes, three members of the *Turandot* gene family, three lysozyme genes as well as several other well known examples such as *Tep1* or *Attacin-D*. In this respect the Asian population shows the opposite pattern. Many of these genes are under-expressed compared to all other populations. We find that this is systematically true for lysozymes (five genes, *q* = 0.0003) and oxidoreductases (18 genes, *q* = 0.0005). No specific functional category was enriched in the list of over-expressed genes for the Asian population.

## Discussion

### Geographic variation of infection resistance

Variation in susceptibility to a generalist parasite can reflect differences in life-history strategy and/or in immune investment between host populations [42–44]. There is some evidence for an effect of latitude on host immune competence where higher species richness in the tropics selects for increased immune investment [14]. Indeed, Tinsley and co-workers found lower susceptibility to the entomopathogenic fungus *B*. *bassiana* in tropical *D*. *melanogaster* populations [15].

Here we assessed susceptibility to *B*. *bassiana* in two tropical and two temperate *D*. *melanogaster* populations. We performed two independent infection experiments, but did not detect a significant difference in susceptibility among host populations. The differences in mortality we observe among infected flies mirror the differences in mortality in control flies. The cause for mortality (and its differences between populations) in the control vials might be the isoparaffinic oils used in the spore and control solutions. These oils have been shown to have insecticidal properties [45] and may pose a stress even in the absence of spores. Contrary to what has been reported previously [15], in our experiments temperate populations performed better than tropical ones, showing lower mortality both in infection and control treatments. This is likely due to their higher general vigor. It is well known that inbreeding can reduce vigor, and even though we used out-crossed populations for our experiments, these were created from inbred lines that have been maintained in the lab for many years. It is possible that the source lines accumulated deleterious alleles during this time and therefore suffer from inbreeding depression. This effect might have been strong enough that subsequent out-breeding between inbred lines could not restore the flies' original vigor. Additionally to the effects of inbreeding there also might be adaptation to the lab environment over time. Flies might have reduced their investment into the ability to cope with infection, as this is costly [3] and flies are rarely immunologically challenged when kept as healthy lab stocks. We would expect that the severity of both effects would correlate with the time of maintenance as inbred lines in the lab. In fact, the African population has been kept as lab cultures for the longest time (collected in 1990). However, all other populations were collected at comparable times: Europe in 1999, Asia in 2002 and America in 2003. So while different times of lab maintenance might contribute to the differences in mortality, it is unlikely to be the main factor. Alternatively, the difference between the tropical and temperate populations could be due to the environment in which they were maintained. However, the temperature at which the flies where maintained (23°C) and at which the infection experiments where performed (25°C) is well within to the physiological optimum range for both temperate and tropical *D*. *melanogaster* [46]. We therefore do not expect an advantage for any population in this respect.

While the differences we observe among our infected flies seem to be explained by differences in general stress resistance one should still not rule out potential differences in the immune system. Phagocytosis, an important component of the early innate immune response in *Drosophila* [47], could act differently in the studied populations. It would furthermore be interesting to see if mortality rates are correlated with parasite abundance. Such measurements could shed light on the actual basis for the observed differences between populations.

The lack of variation in parasite susceptibility among host populations could be due to several reasons. First, we used populations and parasite strains different from Tinsley *et al*. [15]. Second, we worked with *D*. *melanogaster* out-crossed populations instead of F1 crosses generated by mating parental isofemale lines. The use of out-crossed populations, although a closer approximation of natural conditions, introduces more variance in our measurements. In fact each individual is in principle genetically different from the others due to recombination, while in F1 crosses all offspring are virtually identical. This increased variance might reduce our ability to detect differences between populations. Note, however, that we used large population sizes (around 1,000 flies) and have duplicated the out-crossed populations. Furthermore, infection experiments are conducted with large numbers of vials and flies, so we do not expect to lack statistical power to detect possible differences. Another difference is that in Tinsley *et al*. [15] control flies were kept on food containing the antifungal agent Nipagin, while in our case both infected and control flies were reared on Nipagin-free food. The reason for our choice was to assure homogeneity among experimental groups. It is possible that the variability in mortality between host genotypes observed in Tinsley *et al*. [15] reflects, at least partially, a difference in vigor among the *D*. *melanogaster* genotypes used.

*B*. *bassiana*, as a generalist pathogen, is not likely to co-evolve with any host species or population [16]. On the other hand, local adaptation was not found even in the case of *D*. *melanogaster* and its specific parasitoid *Asobara tabida* [48] and, to our knowledge, has not been reported for any *D*. *melanogaster*-parasite combination. The absence of such reports is possibly due to the scarce information available about *D*. *melanogaster* ecology and to the difficulty to detect local adaptation for this species because of its relatively high migration rate [49]. On the other hand, there is evidence of co-evolution between *D*. *melanogaster* and its parasite the sigma virus [50]. This supports the hypothesis that local adaptation is likely to exist between *D*. *melanogaster* and some of its specific parasites in nature, mainly vertically transmitted viral parasites which adapt a higher specificity to their host than horizontally transmitted parasites (such as entomopathogenic fungi). Confirming this hypothesis, genomic studies of *Drosophila* populations revealed that few genes of the immune system are experiencing natural selection [51] except for genes in RNAi pathways involved in resistance to viruses [52]. However, some evidence for local adaptation to abiotic environments has been found in *D*. *melanogaster* populations, for example in terms of a correlation between local climate and ability to enter diapause [53] or between altitude and desiccation resistance [54].

While latitude alone does not appear to be a good predictor of host immune competence in our study system, other environmental variables, such as temperature, humidity or a direct measure of parasite species richness, could be more informative. For example, fly populations coming from locations with a rich bacterial community have been found to be less susceptible to the bacteria *Lactococcus lactis* [55]. Although ecology is important to understand host-parasite interaction [56], we know little about *D*. *melanogaster* in the field and its disease incidence and prevalence, with the exception of Sigma virus [50]. Including more detailed ecological information, such as the prevalence of various pathogens in wild populations of *D*. *melanogaster*, would increase our precision in testing the effect of the environment on host immune investment and other life history traits.

### Transcriptional response to infection

The role of host transcriptional response in determining the out-come of an infection has been highlighted in several studies [22,56–61]. Especially early transcriptional response seems to distinguish susceptible from resistant host genotypes [22,61]. In our study we found that only very few genes were commonly induced eight hours after infection and that the expression change was small. Among the few induced genes were several members of the Cyp450 family. These genes code for enzymes that are important for the detoxification of drugs and toxic compounds, and entomopathogenic fungi are known to produce toxic compounds during infection [62,63]. The metabolism of drugs and toxic compounds usually happens in three steps [64]. The first one is modification, meaning the insertion of a polar group in the target compound. It could happen by oxidation, reduction or hydrolysis. Cyp450 genes can catalyze both oxidative and reductive reactions. The second step is conjugation. In this step the modified toxic compounds are conjugated with negatively charged species such as glutathione, sulfate, glycine, or glucuronic acid. In the third step conjugated compounds are eventually further metabolized and finally actively excreted. Looking from this perspective, one could suggest that also the other induced genes with oxidoreductase activity might be involved in the first phase of toxic compounds metabolism. Additionally, the induced genes with transferase activity might be involved in phase two of toxic compound metabolism. Lastly, we also detect genes involved in transmembrane transport, which might play a role in excretion of toxins. Together these results highlight the importance of detoxification in early response to infection. Overall, we found only very few genes that were regulated differentially between all four populations. Only for the Asian population did we find eleven genes that show expression changes differing from the other three populations. The reason for this might lie in subtle timing differences of the transcriptional response to infection. Of the eleven genes that show expression changes after eight hours in Asia, six show significant changes over all populations 24 hours after infection, with two additional genes being close to the significance threshold (*p* = 0.063 and *p* = 0.075). The direction of expression change in these eight genes after 24 hours perfectly matches the population effect in Asia after eight hours. The Asian population might therefore be reacting slightly faster to the infection than the other populations, resulting in some genes already being regulated differently at an earlier time point.

After 24 hours we find that several thousands of genes are differentially regulated in infected flies compared to controls. This number is much larger than what has been found in previous studies [19,20]. This is not surprising, given the much greater statistical power to detect even subtle changes in gene expression in RNA-seq experiments compared to microarrays. It is unlikely, however, that genes with very small expression changes are of biological significance. We therefore concentrated on those genes with reasonably large expression changes for our further analysis. We found that our list of highly induced genes shows little overlap with the genes found in the microarray studies. Apart from differences in the fly lines used, a further reason could be the differences in infection protocol. While we tried to more closely mimic an infection in a natural setting the aforementioned studies infected flies by shaking them in sporulating fungal material. This is much more drastic than our approach and might not only lead to more severe infections, but also stresses the flies physically. Our infection method may as well introduce a larger variance in the time of infection (that is when spores land on the flies) between flies of the same treatment when compared to the other protocols. These could also be the reasons why the expression changes in our study are quantitatively smaller than what is observed especially in Roxström-Lundquist *et al*. [20]. We find that our most extreme genes are only over-expressed roughly two to five-fold, while Roxström-Lundquist and colleagues report several genes with more than ten-fold over-expression. Both microarray studies find strong induction of the antifungal peptides *Drosomycin* and *Metchnikowin*, which we do not observe. Assuming that the severity of our infections is comparatively small we might well be seeing an earlier stage in infection response at the 24 hour time point compared to the microarray studies.

In our study we find over-expression of genes with catalytic activity including many glucuronosyltransferases, which indicates that the flies are still investing in detoxification processes. We find that the genes that are most strongly down-regulated at the 24 hour time point are all involved in circadian behavior. This is expected as these infected flies where compared to controls for which RNA was extracted 16 hours prior. The expression changes of these genes follow the circadian patterns reported previously [65]. It is interesting to note, however, that other studies in *D*. *melanogaster* have found a functional connection between the circadian rhythm and resistance to infection [66,67] as well as abiotic stressors such as pesticides [68]. Disruption of genes involved in regulation of the circadian rhythm leads to increased susceptibility. Furthermore, it has been found that many genes involved in stress or infection resistance, including Cyp450 genes and glucuronosyltransferases, are regulated in a clock-wise fashion [65,69]. In this context the gene *timeless* shows an interesting behavior. While it follows its expected circadian rhythm overall, it also shows a unique expression pattern in the American population where its repression is much weaker than in all other populations. We can speculate that this difference might have functional consequences in terms of infection response that are specific to the American population.

When we searched for additional genes that were uniquely regulated among the four populations 24 hours after infection we found such genes only in the temperate populations. In the European population two Cyp450 genes show opposite patterns compared to all other populations: *Cyp4e3* is repressed only in Europe, while *Cyp4d14* is repressed everywhere else. This might hint towards a functional equivalence of both genes in the context of response to infection whereas for the European population the functions of the genes are exchanged. For the American flies we found several genes that were uniquely regulated. However, there was no clear pattern in terms of a specific functional class of genes or a specific direction of expression change. It is therefore hard to gauge if and how these genes contribute to infection response and if changes in expression patterns in the American population might have had functional consequences.

Given that our flies originate from four very different populations we would expect genetic divergence to manifest itself transcriptionally. On the DNA level it has been shown that there is appreciable differentiation between populations [70,71]. However, in terms of transcriptional response to infection we do not observe many differences. While at the 24-hour time point we could detect thousands of genes that change expression level, we only found very few genes that show population-specific effects. It therefore seems like expression response is conserved among all tested populations. This makes sense since *B*. *bassiana* is a generalist parasite and we do not expect local co-evolution between host and parasite, which could lead to population differentiation in infection response. On the other hand, we do observe general transcriptional differences between populations. Surprisingly, these differences are only partially in congruence with what has been found on the DNA sequence level, where the African population is most differentiated from all other populations [71]. In our RNA-seq data set the African, Asian and European populations cluster closely together while the American population looks transcriptionally distinct. When we look at the functional classes of genes that show population-specific expression patterns we find many genes that might have changed expression levels as a consequence of adaptation to different food sources such as gustatory and odorant receptors or alpha-mannosidases. Interestingly, many genes with stress and immune related functions are also differentiated between populations. These genes have generally high expression levels in America and particularly low expression levels in Asia. These increased basal expression levels of stress resistance genes in the American population might also be the basis for their outperformance of the other populations in the mortality measurements as it allowed them to better cope with the stresses associated with the experiment. The question remains if this is a consequence of adaptation to a particularly hostile environment in the American population, namely the occurrence of numerous parasite or pathogen species compared to the other populations. Alternatively, other factors such as lab adaptation or inbreeding may also result in decreased expression of these genes in the other populations. It should be noted, however, that both the American and the Asian populations are those that have been kept in the lab for the least amount of time making this explanation unlikely.

## Supporting Information

S1 Table Expression changes 24 hours after infection over all populations. (XLS)

S2 Table Transcriptionally diverged genes at the population level. (XLS)
